# Human Metapneumovirus-associated Atypical Pneumonia and SARS

**DOI:** 10.3201/eid1003.030513

**Published:** 2004-03

**Authors:** Paul K.S. Chan, Ka-Fai To, Alan Wu, Gary M.K. Tse, Kui-Fat Chan, Siu-Fai Lui, Joseph J.Y. Sung, John S. Tam, Brian Tomlinson

**Affiliations:** * The Chinese University of Hong Kong, Prince of Wales Hospital, Shatin, New Territories, Hong Kong Special Administrative Region, China

**Keywords:** human metapneumovirus, respiratory tract infection, postmortem, SARS

## Abstract

Acute pneumonia developed in a previously healthy man during the outbreak of severe acute respiratory syndrome (SARS) in southern China in March 2003. Antibiotic treatment was ineffective, and he died 8 days after illness onset. Human metapneumovirus was isolated from lung tissue. No other pathogen was found. Other etiologic agents should thus be sought in apparent SARS cases when coronavirus infection cannot be confirmed.

Human metapneumovirus (HMPV) is a newly identified member of the family *Paramyxoviridae* (*1*). HMPV infections have been found in all age groups with a wide spectrum of respiratory tract involvement, including a flulike syndrome, bronchitis, bronchiolitis, and pneumonitis (*2*–*5*). We report on a patient who died of acute community-acquired pneumonia, from whom HMPV was the only pathogen identified.

## Case Report

The patient was a 40-year-old Chinese man with previously good health. He smoked but did not use alcohol. He lived in Hong Kong and took frequent short business trips to mainland China. While in Shenzhen in early March 2003, a fever and productive cough with blood-stained sputum developed. He was hospitalized in Shenzhen and given intravenous antibiotics (the details of which were not available). He remained febrile with chills and rigors, night sweating, and progressive dyspnea. He returned to Hong Kong and sought treatment at Prince of Wales Hospital 8 days after the onset of illness. His temperature was 37.3°C, heart rate 120 beats per minute, blood pressure 110/70 mm Hg, and oxygen saturation 93% on room air. No skin rash was detected. Air entry over the right lung had decreased slightly. Chest radiograph showed patchy consolidations in the right middle zone and mild infiltrates in the left lower zone. An electrocardiogram indicated sinus tachycardia with no ischemic changes.

He was admitted to an isolation ward and given supplemental oxygen and cefuroxime,750 mg intravenously every 8 h. Blood tests showed leukocytosis (leukocyte count 14.9x10^9^/L, reference range [RR] 4.0–10.8 x 10^9^/L) with neutrophilia (neutrophil count 13.5x10^9^/L, 91%; RR 41%–73%) and lymphopenia (lymphocyte count 0.5x10^9^/L, 4%; RR 19%–47%). The hemoglobin and platelet count and results of renal and liver function tests were unremarkable. Arterial blood gas while the patient was receiving 2 L of oxygen showed compensated metabolic acidosis (pH 7.39, RR 7.35–7.45; actual bicarbonate 15 mmol/L, RR 22–26 mmol/L; pO_2_ 14.2 KPa, RR 10.0–13.0 KPa).

His condition initially improved with normalization of temperature and heart rate, and a satisfactory level of arterial blood oxygen saturation was maintained until early the next morning, when he reported increasing dyspnea and progressed to shock and severe oxygen desaturation. Before he could be transferred to the intensive care unit, he underwent cardiorespiratory arrest, and aggressive resuscitation attempts were not successful.

## Microbiologic Investigations

On admission (8 days after the onset of illness), sputum and blood samples were taken for routine microscopy, bacterial (including *Legionella pneumophilia* and mycobacteria), and fungal culture. Polymerase chain reactions (PCRs) targeting *Chlamydia pneumoniae*, *Chlamydia. psittaci*, and *Mycoplasma pneumoniae* were performed on the sputum sample.

Nasopharyngeal aspirate was used for virologic investigations. Rapid viral antigen detection by immunofluorescence was performed with commercial assays to identify influenza A and B; parainfluenza 1, 2, and 3; respiratory syncytial virus (RSV); and adenovirus (Dako Diagnostics Ltd, Ely, UK, and Chemicon International, Inc., Temecula, CA). SARS-associated coronavirus (SARS-CoV) was detected by reverse transcription (RT)-PCR by using primers COR-1 (sense) 5′ CAC CGT TTC TAC AGG TTA GCT AAC GA 3′, and COR-2 (antisense) 5′ AAA TGT TTA CGC AGG TAA GCG TAA AA 3′ that had been shown to be specific for the novel coronavirus detected from patients with SARS (*6*). Virus isolation was performed using rhesus monkey kidney (LLC-MK2), human laryngeal carcinoma (HEp-2), Mardin Darby canine kidney (MDCK), human embryonic lung fibroblast, Buffalo green monkey kidney (BGM), and African green monkey kidney (Vero) monolayers. Cell monolayers were examined daily for cytopathic effect. After 14 days of incubation, the growth of influenza A and B, parainfluenza 1, 2, and 3, RSV, and adenovirus was examined by commercial monoclonal antibodies with the immunofluorescence technique. In addition, a hemadsorption test was performed for LLC-MK2 and MDCK monolayers. A shell vial culture system, coupled with monoclonal antibody–based detection, was used for the isolation of cytomegalovirus.

A serum sample taken on admission (8 days after the onset of illness) was evaluated for hantavirus and atypical pneumonia serology, including *Legionella pneumophila; M. pneumoniae; C. pneumoniae* and *psittaci*, influenza A and B; parainfluenza 1, 2, 3; RSV; and adenovirus. In addition, an in-house immunofluorescence assay based on SARS-CoV–infected Vero cells was used to detect SARS-CoV antibodies. This assay has been successfully applied to specimens from patients with SARS. Results of all microbiologic investigations were negative.

## Postmortem Findings

Postmortem biopsy specimens were taken from the lungs, heart, kidney, spleen, small bowel, and muscle. Pulmonary congestion with edema was noted, but hyaline membranes had not formed (Figure A). Interstitial inflammatory cell infiltration was minimal, and intraalveolar organizing lesions were rarely seen, whereas detached atypical pneumocytes were found (Figure B). Atypical multinucleated pneumocytes were observed, but definite viral inclusion was not apparent (Figure C). Fibrin thrombi were frequently observed in small pulmonary arteries and arterioles (Figure D). However, no such thrombotic event was observed in other organs. Central lobular hemorrhagic necrosis was noted in the liver. No myocarditis was observed. Low-grade acute tubular necrosis was seen in the kidneys. Results of examining other tissues were unremarkable. No coronavirus particles were seen on electron microscopy examination of lung cells. The cell culture results were negative, except that a focal refractile rounding cytopathic effect developed 12 days after the left lung tissue sample was injected onto LLC-MK2 cells. This cytopathic effect progressed slowly to cell detachment, and a similar cytopathic effect was observed on other areas of the cell monolayer over the next few days. The cell culture supernatant was positive by an RT-PCR assay based on primers 5′-GAG TAG GGA TCA TCA AGC A-3′and 5′-GCT TAG CTG RTA TAC AGT GTT-3′, targeting the F-gene of HMPV. The nucleotide sequence of the PCR product were identical to the F-gene fragment of HMPV (GenBank accession no. NC 004148) (*1*). The cell culture supernatant was passaged to LLC-MK2 cells, and the same cytopathic effect was observed after 8 days of incubation. HMPV particles were shown from the passaged cell culture by electron microscopy. To ascertain the presence of HMPV infection in this patient, the serum sample taken on admission (8 days after the onset of illness) was retrieved for HMPV antibody detection with the immunofluorescence technique, based on LLC-MK2 cells infected by the isolated HMPV. An antibody titer of 1:80 was detected.

**Figure:**
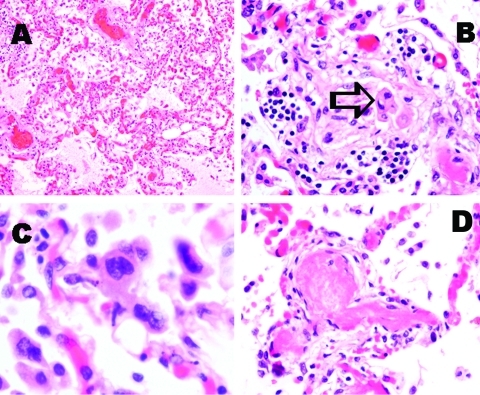
Pathologic findings of lung tissue sections. A: Pulmonary congestion and edema (H&E stain, original magnification x100). B: A mild degree of interstitial lymphocytic infiltration. Intra-alveolar organizing exudative lesion was occasionally found. Detached atypical pneumocytes indicated by arrow (H&E stain, original magnification x200). C: Atypical multinucleated pneumocytes were occasionally identified. Definite viral inclusion was not apparent (H&E stain, original magnification x400). D: Fibrin thrombi were frequently noted in small pulmonary arteries and arterioles (H&E stain, original magnification x200).

## Conclusions

HMPV is a recently identified human virus associated with respiratory tract disease. While the full range of clinical features is still being elucidated, several cases of severe respiratory tract infections have been reported. Boivin et al. (*2*) reported that 3 of 12 infected young children <5 years of age required intensive care, and a child with acute leukemia died subsequently. In that study, one of the six infected adult patients (15–65 years of age) also required intensive care. In the same study, 2 of 10 infected elderly patients (ages >65 years) died; 1 also had leukemia and the other had Alzheimer’s disease. However, none of these patients had an autopsy performed. This report is the first in which HMPV was the only pathogen identified from postmortem specimens from a patient with a fatal respiratory tract disease. Our patient had a history of good health and no evidence to suggest that his death was a result of exacerbation of underlying cardiac or pulmonary conditions. Coinfection has been found in patients with HMPV-associated respiratory tract disease (*2*) and has been suggested to be a factor influencing clinical outcome (*7*). In our patient, no evidence of infection with a copathogen could be demonstrated. Although the patient had died before a late convalescent-phase serum sample could be obtained for a confident exclusion of SARS-CoV infection by serologic testing, the nasopharyngeal aspirate collected on admission and the postmortem lung tissue were negative for SARS-CoV by RT-PCR. The postmortem histologic findings on lung tissue of this patient were different from the classic picture of virus-associated interstitial pneumonitis. In addition to the minimal inflammatory cell infiltration, multinucleated giant cells that might represent viral cytopathic effect were only occasionally seen. Rather, the predominant histologic finding was the presence of fibrin thrombi in small pulmonary arteries and arterioles. This thrombotic event was confined to lung tissue and was not found in other tissues. Thrombotic vasculopathy has been observed in infectious diseases caused by HIV, cytomegalovirus, and herpes viruses (*8*,*9*). The clinical picture usually resembles thrombotic thrombocytopenic purpura. However, our patient’s condition appeared distinct. He showed no serologic or morphologic evidence of other infective agents. He was negative for HIV antibody and did not fulfill the diagnosis of thrombotic thrombocytopenic purpura. The pathogenic event that led to the formation of thrombi was unclear.

Our patient’s signs and symptoms, together with his history of travel to southern China, fulfilled the World Health Organization criteria for a probable case of SARS, but no evidence of coronavirus infection could be found. The overall pathologic features of this case were distinct from those in the series of SARS patients that we had examined and that were reported by others (*10*). In SARS patients, coronavirus particles were seen by electron microscopy in lung cells of most cases, and the lung injuries consistently exhibited features of diffuse alveolar damage. Distinctive airspaces or small airway lesions resembling bronchiolitis obliterans organizing pneumonia were also detected. These SARS-associated pathologic features were not observed in this patient, suggesting a different cause of death. In this patient, HMPV infection was confirmed by virus isolation from lung tissue. Although the possibility of HMPV being a bystander could not be totally excluded, a role of HMPV in severe respiratory tract disease should not be discounted, particularly in SARS patients that do not show evidence of coronavirus infection. HMPV has also been detected in five of the six SARS patients living in Canada; four of them were coinfected with coronavirus (*11*). The index patient of that cluster had stayed in Hong Kong before the onset of illness. The patient described in this report was unlikely to have a direct link to the outbreak of SARS in our hospital. This patient was admitted around the same time that an outbreak of SARS began in our hospital. That outbreak mainly involved staff working at the Accident and Emergency Department and the index ward where a SARS patient had been treated. The index ward is located on a different floor from where the patient described in this report was isolated. In addition, neither fever nor respiratory illness developed in any of the healthcare workers involved in taking care of this patient in the next 2 weeks, and we were not aware of any spread to his family members.

Although HMPV and SARS-CoV might differ in transmission efficiency, their role and clinical outcome in acute respiratory tract disease need to be distinguished. This distinction is particularly important for analyzing the illness and death associated with the worldwide outbreak of SARS. Patients fulfilling the clinical criteria for a probable case of SARS might be infected by organisms other than coronavirus.
